# Union is strength: the combination of radiomics features and 3D-deep learning in a sole model increases diagnostic accuracy in demented patients: a whole brain 18FDG PET-CT analysis

**DOI:** 10.1097/MNM.0000000000001853

**Published:** 2024-04-18

**Authors:** Alberto Bestetti, Barbara Zangheri, Sara Vincenzina Gabanelli, Vincenzo Parini, Carla Fornara

**Affiliations:** a Department of Clinical and Community Sciences, State University of Milan, Milan,; b Nuclear Medicine Department, MultiMedica Hospital,; c Radiation Oncology Department, MultiMedica Hospital and; d Division of Neurology, MultiMedica Hospital, Sesto San Giovanni, Italy

**Keywords:** combined model, deep learning, dementia, FDG PET, radiomics

## Abstract

**Objective:**

FDG PET imaging plays a crucial role in the evaluation of demented patients by assessing regional cerebral glucose metabolism. In recent years, both radiomics and deep learning techniques have emerged as powerful tools for extracting valuable information from medical images. This article aims to provide a comparative analysis of radiomics features, 3D-deep learning convolutional neural network (CNN) and the fusion of them, in the evaluation of 18F-FDG PET whole brain images in patients with dementia and normal controls.

**Methods:**

18F-FDG brain PET and clinical score were collected in 85 patients with dementia and 125 healthy controls (HC). Patients were assigned to various form of dementia on the basis of clinical evaluation, follow-up and voxels comparison with HC using a two-sample Student’s *t*-test, to determine the regions of brain involved. Radiomics analysis was performed on the whole brain after normalization to an optimized template. After selection using the minimum redundancy maximum relevance method and Pearson’s correlation coefficients, the features obtained were added to a neural network model to find the accuracy in classifying HC and demented patients. Forty subjects not included in the training were used to test the models. The results of the three models (radiomics, 3D-CNN, combined model) were compared with each other.

**Results:**

Four radiomics features were selected. The sensitivity was 100% for the three models, but the specificity was higher with radiomics and combined one (100% vs. 85%). Moreover, the classification scores were significantly higher using the combined model in both normal and demented subjects.

**Conclusion:**

The combination of radiomics features and 3D-CNN in a single model, applied to the whole brain 18FDG PET study, increases the accuracy in demented patients.

## Introduction

Alzheimer’s disease (AD) is known to be the most common neurodegenerative disease. It has been estimated that over 46 million people live with dementia worldwide, increasing to 131.5 million by 2050 [1]. Therefore, there is an urgent need for biomarkers to be used for screening, diagnosis, prognosis and therapy response to control the societal impact of the disease. FDG PET imaging offers valuable insights into the functional alterations of the brain by measuring regional cerebral glucose metabolism. Radiomics and deep learning techniques provide complementary approaches for analyzing FDG PET images, enabling quantitative and automated evaluation [2]. Radiomics has recently shown interesting applications in the neurology field, highlighting promising results in differential diagnosis [3] among causes of cognitive impairment and in the prediction of the conversion from mild cognitive impairment (MCI) to Alzheimer dementia. A recently published review of the literature [4] analyzed the manuscripts and articles of the last 5 years from the PubMed, Scopus and Embase databases. All studies concerning discrimination among AD, MCI and healthy older people performing radiomics analysis through machine and deep learning were included. In 14 out of 15 studies, regions of interest were extracted from MR images, while only in [5], Aβ PET was used to detect the accumulation of the beta-amyloid protein in the brain. For the identification of AD from healthy controls (HCs) with the radiomics features of Aβ PET images, the authors obtained an AUC = 0.93 with the standard machine learning method, while in all other studies using MRI, the AUC values ranged from 0.72 to 0.93. The segmentation of the MRI/PET image of the brain was aimed at extracting one or more anatomical areas of interest from which radiomic features were then calculated. Moreover, these quantitative features have the potential to differentiate various types of dementia, predict disease progression and evaluating treatment response [6–8]. Yupeng *et al*. [8] assessed FDG PET and clinical cognitive scales in AD, MCI and HC subjects, from Alzheimer’s Disease Neuroimaging Initiative (ADNI) cohorts. They determined the brain region of interest involved (ROIs), comparing, by a two-sample Student test, AD patients and HC and used them for radiomics analysis. Pearson’s correlation coefficients were regarded to select effective features associated with the clinical cognitive scales. Using a support vector machine as a test classifier, an accuracy of 91.5% was obtained for classifying AD vs. HC. Deep learning techniques when applied to FDG PET images can learn complex patterns and relationships directly from the data without relying on predefined features. Convolutional neural networks (CNNs) can be trained to classify dementias, identify biomarkers and detect subtle changes in FDG uptake patterns, offering high accuracy and efficiency in the evaluation process [9–12]. The majority of these studies for AD diagnosis were conducted on MRI scans. Sotoudeh *et al*. [3] used multiple deep 3D-CNN on different local image patches to learn the discriminative features of MRI and FDG PET images, obtained from the ADNI dataset. The classification performance for AD/HC diagnosis was 93.3%. In a recently published study [13], Bestetti *et al*. compared radiomics and 3D-deep learning (CNN) in the evaluation of 18F-FDG PET whole brain images in patients with dementia and normal controls. They concluded that radiomic features seem to be more specific than CNN to distinguish patients with and without dementia. The aim of the present study was to improve the accuracy of the individual models by adding, in the fully connected layer of the 3D-CNN, the features obtained from whole brain 18FDG PET/CT Radiomics analysis.

## Materials and methods

### Image acquisition

All patients who underwent 18F-FDG PET/computed tomography (CT) brain scans at MultiMedica Hospital were in a resting state, following ≥4–6 h fast, and had a measured blood glucose level <11.0 mmol/l at the time of the study. A 222–296 MBq injection of 18F-FDG was administered intravenously under standardized conditions (in a quiet, dimly lit room with the patient’s eyes open). A 10-min three-dimensional (3D) brain emission scan was acquired at 30–45 min postinjection with a PET scanner (Siemens Biograph 8 HD PET/CT; Siemens, Germany). During the scanning procedure, the patient’s head was immobilized using a head holder. Attenuation correction was performed using low-dose CT prior to the emission scan. Following corrections for scatter, dead time and random coincidences, PET images were reconstructed using OSEM iterative reconstruction providing 110 contiguous transaxial slices of 3.3-mm-thick spacing.

### Image preprocessing

All the original digital imaging and communications in medicine data were converted into NIfTI formatted files. [18F]FDG PET images of patients and controls were normalized to the optimized [18F]FDG PET template [14], using SPM12 (https://www.fil.ion.ucl.ac.uk/spm/) implemented in MATLAB R2023b. They were then scaled to the global mean of the activity within the brain and finally smoothed with an isotropic 3D Gaussian kernel (8 mm FWHM), accordingly to the validated pipeline proposed for our single-subject SPM-based analysis [14]. This kind of smoothing is required for the random field theory to be applicable. It is also effective in reducing the number of multiple comparisons to be performed. The normalized images had a spatial dimension of 79 × 95 × 69 with voxel sizes of 2 × 2 × 2 mm^3^, in order to obtain rotationally invariant texture features.

**Fig. 1:**
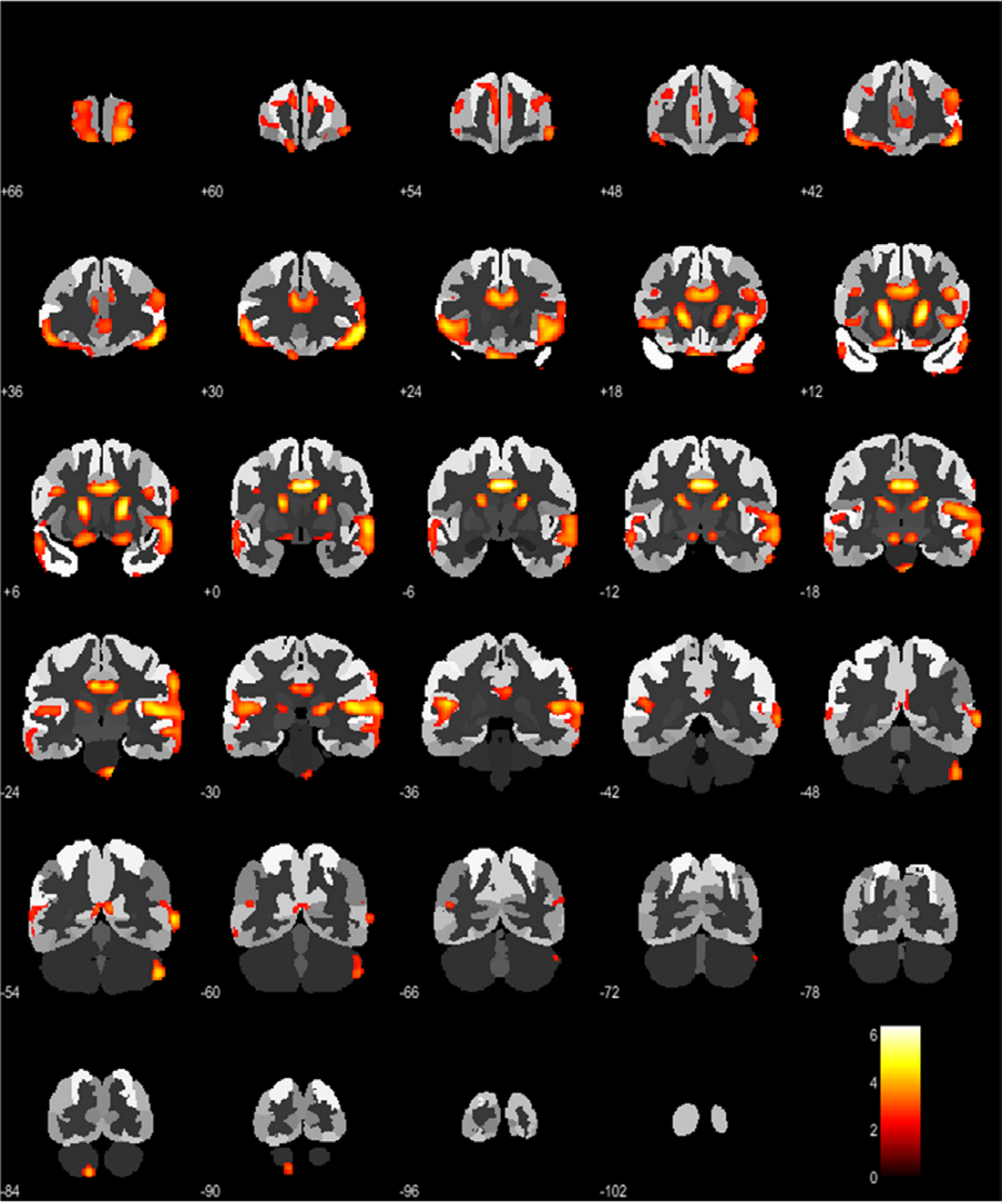
Coronal images show the result of the two-sample Student’s test to assess the difference between AD patient and HC group. Regions of significant decreases in FDG uptake, are overlapped on the standard SPM template. It shows an example of a patient with frontotemporal, behavior variant, dementia. AD, Alzheimer’s disease; HC, healthy controls.

The SPM statistical voxel-wise procedure consists of a *t*-test, in which a single individual is compared with a dataset of HC, entering age as a covariate. This statistical comparison provides *t*-scores for each brain voxel [15]. SPM *t*-maps were generated from this statistical comparison in order to identify eventual brain areas of significant hypo metabolism (*P* < 0.01). Only clusters containing more than 100 voxels were considered to be significant [15]. Two neuroimaging experts visually inspected all the SPM *t*-maps in order to confirm the presence of hypo metabolism patterns compatible with neurodegenerative processes. Figure 1 shows an example of a patient with frontotemporal, behavior variant and dementia.

### Feature extraction

Unlike other authors [8] who used several ROIs positioned in specific areas of the brain, we preferred to employ the entire brain volume as reference (VOI). Image intensity level was discretized into 32 bins.

We computed 79 first-order statistic features (morphology, statistical, histogram and intensity-histogram features), describing the distribution of voxel intensities within the ROI mask and, to quantify intra-ROI heterogeneity, we extracted 136 3D textural features by analyzing the gray level cooccurrence matrix (GLCM), run-length matrix (GLRLM), size-zone matrix (GLSZM, GLDZM) and gray level dependence matrix (NGLDM), neighboring gray-tone difference matrix (NGTDM).

The standardized environment for radiomics analysis package [16] (a Matlab-based framework) was used for this purpose, in which features are consistent with the guidelines of image biomarker standardization initiative. This package has been assessed in multicenter standardization studies [17,18] to ensure reproducibility of the features.

### Statistical analysis

For radiomic features analysis, the minimum redundancy maximum relevance (MRMR) method was used to address the dimensionality problem [19]. The MRMR algorithm finds an optimal set of features that is mutually and maximally dissimilar and can represent the response variable effectively. The algorithm minimizes the redundancy of a feature set and maximizes the relevance of a feature set to the response variable. Correlation analysis among the selected features was then performed in terms of the Pearson correlation coefficient, selecting only those with a correlation inferior to 30%. The final set of features was used to train. For this purpose, the dataset was split into 90% training and 10% validation data. A binary classification model was built using the Matlab function ‘featureInputLayer’ which inputs feature data to a neural network and applies z-score normalization as detailed in our previous work [13]. The endpoint was to discriminate between normal controls and demented patients. The accuracy of the trained model was then assessed on the testing data (40 subjects).

### Convolutional neural network architecture

Convolutional neural networks are the basis of deep learning methods which excel at pattern recognition. The CNN is designed to better retain and utilize the structural information among neighboring voxels and to require minimal preprocessing by directly taking 3D images as inputs. Structurally, a CNN is a sequence of layers and each layer of the CNN transforms one volume of activations to another through a differentiable function. A typical CNN consists of three types of neural layers: convolutional layers, pooling layers and fully connected layers. The convolutional layers are interspersed with pooling layers, eventually leading to the fully connected layers. The convolutional layer takes the voxels of a small patch of the input images, called the local receptive field and then utilizes various learnable kernels to convolve the receptive field to generate multiple feature maps. A pooling layer performs the nonlinear down-sampling to reduce the spatial dimensions of the input volume for the next convolutional layer. The fully connected layer inputs the 3D feature map to a 1D feature vector. The major issue in training deep models is over-fitting, which arises from the gap between the limited number of training samples and a large number of learnable parameters. Batch normalization it is one of the most used methods to remedy it. The scheme of the CNN model was reported in detail in the previous study [13]. The model was subjected to training, validation and testing using the same Radiomics dataset.

### Combined model

In the hope of increasing the diagnostic accuracy of individual models, we added in the fully connected layer of the 3D-CNN the final set of features. The code was in-house developed using MATLAB 2023b environment.

#### Model scheme

**Fig. 2:**
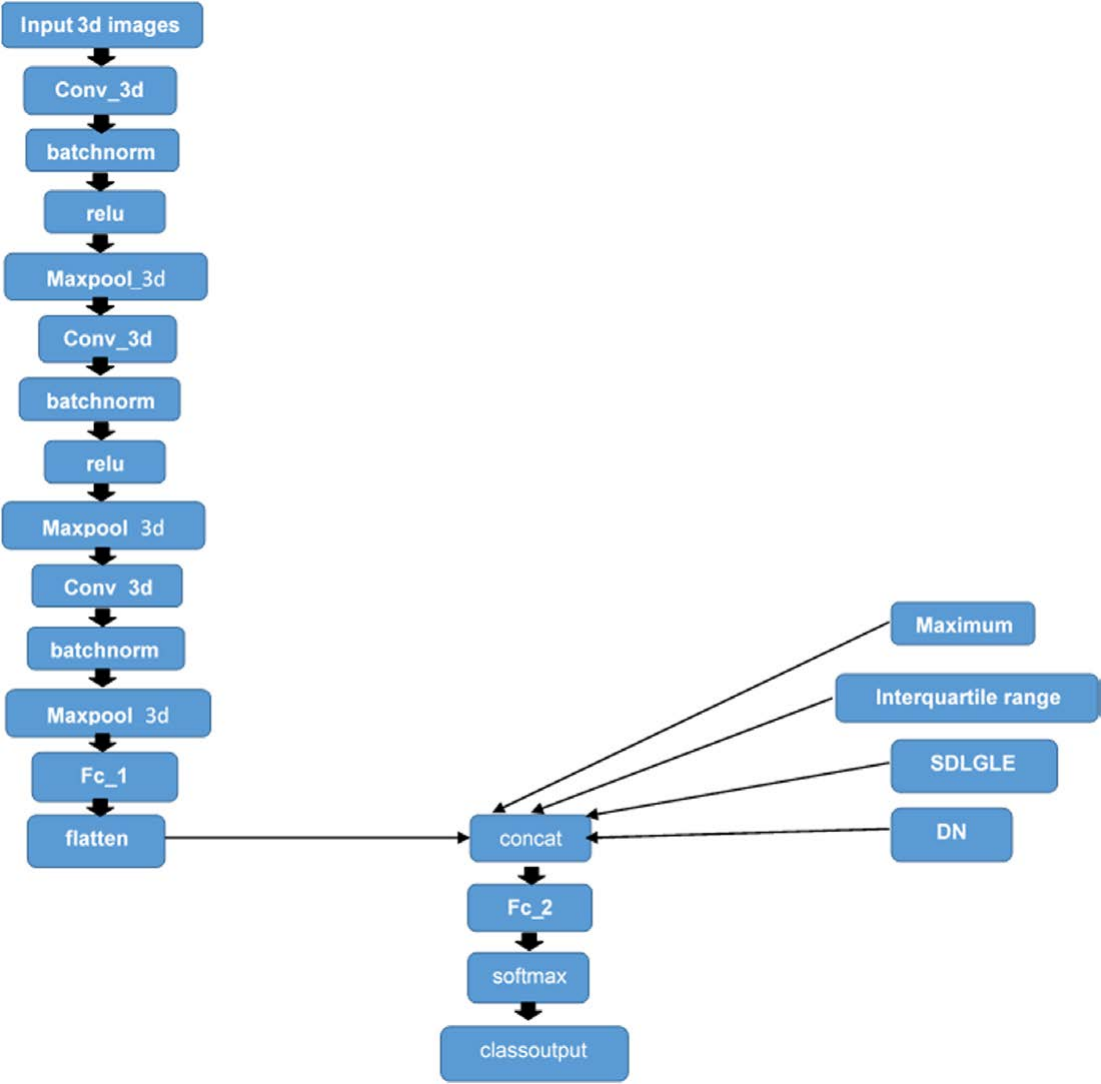
Combination of 3D-CNN model and radiomics features.

1. For the image input, an image input layer with a size matching the input data (79 × 95 × 69).
2. For the feature input, a feature input layer with size matching the number of input features (four features).
3. For the image input branch, was specified a convolution, batch normalization, and ReLU layer block, where the convolutional layer has 16 5-by-5 filters.
4. To convert the output of the batch normalization layer to a feature vector, was included a fully connected layer of size 50.
5. To concatenate the output of the first fully connected layer with the feature input, was flattened the ‘SSCB’(spatial, spatial, channel, batch) output of the fully connected layer so that it has the format ‘CB’ using a flatten layer.
6. Was concatenated the output of the flatten layer with the feature input along the first dimension (the channel dimension).
7. For classification output, was included a fully connected layer with output size matching the number of classes (normal and demented), followed by a softmax and classification output layer.
8. Was created a layer array containing the main branch of the network and convert it to a layer graph. The resulting graph is shown in Fig. 2.
9. We used 90% of the dataset for the training cohort, and 10% of the dataset for the validation cohort. We tried different ratios: 90–10%, 80–20%, 70–30% and picked that it gave the best performance result. The accuracy of the three models (radiomics, 3D-DL and the combined one) was tested on 40 new patients, 20 normal and 20 demented. The accuracy and the classification scores (the classification score represents the posterior probability that the individual case belongs to the class of normal or pathological) were compared using a Student’s *t*-paired test (statistically significant *P* < 0.05).

### Patient population

The study was approved by the ethics committee of MultiMedica Hospital, Sesto San Giovanni, Italy. All patients of MultiMedica Hospital provided written informed consent. The data used in this study included 125 HC obtained from the AIMN website (aimn.it). The AIMN represents the Italian scientific reference for nuclear medicine and molecular imaging activities. This database comprised subjects aged between 23 and 84 years (mean: 64.8 ± 11.3), 53% males. These subjects were selected because they were characterized by absence of global cognitive impairment, as assessed by a MMSE score ≥28, and were cognitively normal after an average 4-year clinical follow-up. From a clinical cohort referred to the Departments of Neurology and Nuclear Medicine Unit of MultiMedica Hospital (Milan, Italy), we retrospectively selected 85 consecutive patients diagnosed with probable dementia based on [18F] FDG PET and clinical examination results, namely, 38 cases diagnosed with probable AD, 2 MCI, converted to AD [20], 25 with probable Lewy body dementia (DLB) [21], 20 fulfilling current clinical criteria for frontotemporal dementia [22]. All patients had to have a confirmed neurological diagnosis after at least 2-year follow-up. The clinicians, unaware of PET results, were the same between the training and testing sets. The diagnosis was further confirmed by comparison with HC, using Student’s *t*-test as described in ‘Image Preprocessing paragraph’. The characteristic metabolic patterns present in various causes of dementia have been reported in several previous studies [23,24]. This group comprised subjects aged between 56 and 88 years (mean: 73.9 ± 6.8), 50 males (59%). The mean Mini-Mental State Examination score was 20.2. The test group was constituted by 20 normal subjects (mean age: 62.1 ± 16.1, 50% males), who have performed a total body 18FDG PET scan for presumed or confirmed oncological pathology, without a previous history of neurovascular diseases, whose brain PET was normal compared with HC, and by 20 demented patients: 6 AD, 6 FT, 2 MCI, 4 DLB and 2 affected by Corticobasal degeneration (mean age: 75.5 ± 7.5, 41% males). The mean MMSE was: 19.4 ± 4.9. The selection criteria were the same used for the enrollment of training patients. The demented group was significantly older than the healthy group (*P* = 0.005) (Table 1), while no significant difference was found when comparing the ages of normal subjects in the training group vs. control group (53% vs. 50%), while as regards the demented population, the prevalence of males was lower in the test group (40% vs. 58%, *P* < 0.001).

**Table 1:** Probability score for a subject to be normal or pathological for various types of dementia

	Demented							HC				
Pt	Cnn	Rad	Comb	Sex	Age	MMSE	Diagnosis	Cnn	Rad	Comb	Sex	Age
1	97.9	85.6	99.9	F	73	23	AD	93.2	96.9	99.8	M	37
2	99.7	99.8	100	F	85	16	MCI	96.5	97.7	100	F	70
3	89	97.6	99.8	F	80	17	DLB	96.3	97.3	100	F	71
4	100	100	100	M	69	20	DLB	87.9	97.5	99.8	M	67
5	99.4	99.9	100	F	79	17	CB	57.3^a^	95.9	99	M	69
6	95.2	97.2	99.9	M	58	20	FT	97.4	97.7	99.8	M	55
7	94.5	96.3	99.8	F	72	16	AD	89.5	97.1	99.7	F	84
8	96.9	98.6	100	F	82	23	AD	83.1	97.4	99.7	F	72
9	1000	100	100	M	76	22	DLB	69.5	96.9	99.9	M	36
10	98.8	99.2	100	M	69	23	AD	75.2	97.1	100	F	73
11	99.5	99.5	100	F	71	21	FT	50.7^a^	93.2	99.9	F	67
12	83.6	80.7	99.8	F	87	30	MCI	87.2	97.3	100	M	27
13	90.4	84.8	99.8	F	76	15	AD	98.6	95.3	99.8	F	73
14	98.6	92	99	M	78	5	FT	84.6	97.9	100	F	78
15	96.8	99.5	100	F	66	19	FT	50.5^a^	95.3	99.8	M	62
16	100	99.9	100	M	73	20	FT	59.6	96	100	F	76
17	100	99.3	100	M	84	17	DLB	95.6	97.4	100	M	34
18	99.7	99.6	100	M	68	18	AD	95	98	100	M	59
19	89.4	99.8	99.7	F	86	19	CB	79.1	97.6	100	M	64
20	99.9	98.2	100	F	77	23	FT	97.7	98	100	F	69
mean	97.8	97.3	99.9	M (40%)	75.5	19.2		87.1^b^	97.3	99.9	M (50%)	62.1
SD	4.92	4.62	0.28		7.51	4.83		11.4^b^	1.15	0.3		16.1

AD, Alzheimer dementia; CB, corticobasal degeneration; Comb, CNN + rad; DLB, Lewy body dementia; FT, frontotemporal dementia (bv = behavioral variant); HC, healthy controls; MCI, mild cognitive impairment; Rad, radiomics.

a Three subjects classified as pathological by CNN.

b Values obtained after have omitted those three subjects.

## Results

### Model analysis

The MRMR analysis identified 21 features, equal to 10% of the total. The correlation analysis identified four noncollinear features [13] which were used to train. Maximum and Interquartile range are simple first-order statistics features, while dependence counts nonuniformity (DN) and low dependence low gray level emphasis (SDLGLE) belong to the category of gray level dependence matrix (GLDM), high-order features. A GLDM quantifies gray level dependencies in an image, that is, the number of connected voxels within distance δ that are dependent on the center voxel. A neighboring voxel with gray level *j* is considered dependent on center voxel with gray level *i* if |*i* − *j*|≤α. In a GLDM *P (i, j*) the (*i, j*) ^th^ element describes the number of times a voxel with gray level *i* with *j* dependent voxels in its neighborhood appears in image. Specifically, DN measures the similarity of dependence throughout the image, with a lower value indicating more homogeneity among dependencies in the image, while SDLGLE measures the joint distribution of small dependence with lower gray level values [25].

The overall validation accuracy of all three models was 100%. All models correctly identified the 20 demented patients that constitute the test group. As shown in Table 1, the scores indicating the likelihood of disease in each subject were similar using Radiomics and the CNN model, but they were significantly higher with the combined method compared with the other two (Table 2). While radiomics and the combined model correctly identified all normal subjects, the CNN model classified three subjects as pathological with a likelihood of disease just over 50% (specificity = 85%). Omitting those three subjects, the probability score was still significantly higher with radiomics compared with CNN (Table 2), and was also significantly higher when the combined model was compared with Radiomics (Table 2).

**Table 2:** Comparison of the probability score between the three models

	Demented	HC
	*P*	*P*
CNN vs. RAD	NS	0.002
CNN vs. COMB	0.004	0.0004
RAD vs. COMB	0.013	<0.0001

## Discussion

In the current AD diagnostic criteria, cerebrospinal fluid (CSF), namely, CSF amyloid_ 42 (A_ 42) or A_ 42/40, phosphorylated tau and total tau levels and PET biomarkers, that is, amyloid PET and tau PET, were often included. However, these biomarkers are affected by some limitations. In particular, CSF biomarkers can be assessed only by a lumbar puncture, which is considered quite an invasive procedure, and amyloid PET and tau PET are expensive and not always available tools. Therefore, growing efforts are being made by researchers in order to identify reliable, easily available and noninvasive AD biomarkers. In this regard, radiomics and CNN-DL might be an interesting option [4]. Radiomic features which are extracted directly after segmentation from the area of interest, can be broadly categorized into five classes: first-order features, shape-based features, texture features, wavelet-based features and deep learning-based features. First-order features, or statistical features, were used to describe the basic statistics of the voxel intensity distribution within a ROI, such as the mean, median, variance, skewness and kurtosis. Shape-based features describe the geometric properties of the ROI, such as the volume, surface area and compactness. Texture features capture the spatial distribution of voxel intensities within the ROI and provide information on the heterogeneity, coarseness and complexity of the tissue. Texture features are extracted using different methods, such as the GLCM, GLRLM, GLSZM, GLDM and NGTDM, as accomplished in all reported studies. It is a matter of discussion whether CNNs or radiomics are preferable in the field of medical imaging and neuroradiology. A major incentive for favoring radiomics is the black-box nature of CNNs. The basis of the output of the CNN is not always readily explicable. In a radiomics pipeline, features are extracted based on predefined mathematical equations, designed by image analysis experts. However, not every radiomics pipeline is easily comprehensible either. The interpretation of second and higher-order features can be quite challenging. Furthermore, the model itself may not be transparent. If a feed-forward neural network is used, for example, it can be difficult to determine how much each feature contributed to the output [26].

The aim of the present work is twofold: to confirm on a larger population the results previously obtained [13] and to verify whether the combination of the two models is more accurate, overcoming the limits of individual models. In both studies the analysis was conducted on the whole brain, using the same deep learning model and radiomic features. The choice was dictated by two reasons. First to avoid the mispositioning of the ROIs due to the low spatial resolution of the PET. When placing the boundaries of the region or volume of interest, it needs to be ascertained that these boundaries are drawn correctly, consistently and in a fashion that is appropriate for the research question that is to be addressed. Regions of interest should be placed by individuals well-versed in image interpretation. Secondly, although each type of dementia has specific areas involved, since the brain is an interconnected whole, an integral approach could be preferable. The most specific radiomic parameters in order to distinguish normal from pathological subjects are probably the two high-order features thanks to the possibility of assessing the homogeneity or inhomogeneity of voxel count distribution. In fact, our 3D-DL model, compared to Radiomic features, classified incorrectly three normal subjects as pathological with a likelihood of disease just over 50% (specificity = 85%). In order to overcome the limits of each model, and this is the novelty of the present work, we have integrated the four features obtained from radiomics and the fully connected layer of the 3D-CNN. The probability scores resulted significantly higher when the combined model was compared with radiomics both in normal subjects (*P* = 0.0003) and demented patients (*P* = 0.012).

## Limitations

There are several caveats when conducting radiomics and DL research. The small size of our demented group was only partially compensated by the high-quality images obtained with homogeneous acquisition protocols and reconstruction techniques. Furthermore, radiomic features are notorious to be significantly sensitive to batch effect (variability in imaging acquisition parameters in different centers, scanner models and reconstruction settings) [27,28]. A number of harmonization methods have been proposed to correct for the batch effect to generate robust and reproducible models. But these methods are mandatory when using multicenter datasets [29], while our patients come from only one. The PET scanners used to acquire the controls taken from the reference database are similar to those used in our department.

## Conclusion

The novel approach based on a combination of high-order radiomics features extracted from standardized 18F-FDG PET whole brain images and 3D-deep learning seems to be more accurate than single approaches to distinguish patients with and without dementia. It can be an actual strength that can potentially add to the interpretation of experts.

## Acknowledgements

The authors wish to recognize the valuable human and professional contribution of technical and nursing staff of Nuclear Medicine Department, especially the role of technical chief, Carmelo Torrito. We are also grateful to Mr. Giovanni Maria Bestetti for his invaluable assistance in this study.

All authors contributed to the study conception and design. Material preparation, data collection and analysis were performed by A.B., B.Z., S.V.G., V.P. and C.F. The first draft of the manuscript was written by A.B. and all authors commented on previous versions of the manuscript. All authors read and approved the final manuscript.

The datasets generated and analyzed during the current study are available from the corresponding author upon reasonable request.

### Conflicts of interest

There are no conflicts of interest.
